# Validity and Reliability of the Attachment Insecurity Screening Inventory (AISI) 2–5 Years

**DOI:** 10.1007/s12187-015-9322-6

**Published:** 2015-08-14

**Authors:** I. B. Wissink, C. Colonnesi, G. J. J. M. Stams, M. Hoeve, J. J. Asscher, M. J. Noom, N. Polderman, M. G. Kellaert-Knol

**Affiliations:** Department of Child Development and Education, University of Amsterdam, Amsterdam, The Netherlands; GGzE Department of Forensic Child and Adolescent Psychiatry, Eindhoven, The Netherlands; Basic Trust (organization of specialists in education, attachment and adoption/foster care), Haarlem, The Netherlands; Faculty of Social and Behavioural Sciences, Department of Child Development and Education, Forensic Child and Youth Care, P.O. Box 15776, 1001 NG Amsterdam, The Netherlands

**Keywords:** Attachment, Instrument development, Early childhood, Validation study, Preschoolers

## Abstract

The Attachment Insecurity Screening Inventory (AISI) 2–5 years is a parent-report questionnaire for assessing attachment insecurity in preschoolers. Validity and reliability of the AISI 2–5 years were examined in a general sample (*n* = 429) and in a clinical sample (*n* = 71). Confirmatory factor analysis (CFA) confirmed a three-factor model of avoidant, ambivalent/resistant and disorganized attachment, and one higher-order factor of total attachment insecurity. Multi-group CFA indicated measurement invariance across mothers and fathers, and across the general and clinical population sample. Reliability coefficients were generally found to be good. We found partial support for convergent validity in associations between AISI-scores and observed attachment (AQS). Concurrent validity was supported by associations between AISI-scores and observed parental sensitivity (MBQS) and parent-reported psychopathology (SDQ). Finally, the AISI discriminated well between children from the general and from the clinical sample. We argue that both research and practice could benefit from the AISI as there is now a prospect of quickly, reliably and validly screening for attachment insecurity in pre-school aged children. Based on this information, help can be offered timely and, subsequently, the prevention of attachment related problems of children can be strengthened.

One of the most important developmental challenges in infancy and early childhood is building secure child-parent attachment relationships, as attachment security provides a secure base from which young children can explore the physical and social environment. There is, however, empirical evidence showing that 38 % of the children from the general population have insecure attachment relationships (Van IJzendoorn et al. 1999). This percentage is even higher in clinical samples (Dozier et al. 2001; Van IJzendoorn et al. 1999). The present study aims to examine the validity and reliability of the Attachment Insecurity Screening Inventory (AISI) 2–5 years, which is a parental report questionnaire to screen for attachment insecurity in children from 2 to 5 years of age.

According to Bowlby (1988, p. 26), attachment concerns ‘any form of behaviour that results in a person attaining or maintaining proximity to some other clearly identified individual who is conceived of as better able to cope with the world’. The forms of attachment security and insecurity are determined by the strategy of maintaining proximity to the caregiver and are responses to the sensitivity of the parent (see meta-analyses by Atkinson et al. 2000; De Wolff and Van IJzendoorn 1997; Van IJzendoorn and De Wolff 1997). Secure children (Type B) use their consistently sensitive parent(s) both as a secure haven and secure base, finding a balance between ‘proximity seeking’ and ‘exploration of the environment’ (Ainsworth et al. 1978; Cassidy and Marvin 1992; Main and Solomon 1986, 1990). Insecure children do not show this secure response pattern, but show one of three insecure patterns: Insecure-Avoidant (Type A), Insecure-Ambivalent or resistant (Type C) or Insecure-Disorganized (Type D). Insecure-Avoidant children (Type A) minimize attachment behaviors, which is an insecure but still organized strategy to keep proximity to a consistently insensitive and rejecting parent. Insecure-Ambivalent or resistant children (Type C) maximize attachment behaviors, which is an insecure-organized strategy to keep proximity to a parent who is inconsistently sensitive. Insecure-Disorganized children (Type D) do not have an organized strategy to keep proximity to their caregiver. They display seemingly non-goal directed and disoriented behavior in infancy, experiencing a conflict between seeking proximity and fearing to approach the caregiver. Disorganized children may show combinations of insecure strategies, such as avoidance and ambivalence/resistance, or use controlling strategies in response to their parents during preschool age. These controlling behaviors may be punitive and aggressive or care giving (being overly solicitous and nurturing with the parent) in order to guide the parent’s behavior (Vallance 2004). Disorganized or controlling attachment is considered to be a response to extremely insensitive caregivers, who are frightening, hostile, and abusive (punitive subtype) or frightened and helpless (caregiving subtype). It has been shown that parent–child interaction is most disrupted in preschoolers classified as disorganized compared with children designated as ambivalent/resistant or avoidant (Moss et al. 1998), which concurs with research findings showing that disorganized children should be considered as the most insecure (O’Connor and Zeanah 2003).

Attachment insecurity constitutes a serious risk for the development of psychopathology. That is, an insecure child-parent attachment relationship has been shown to be associated with both internalizing (Brumariu and Kerns 2010; Colonnesi et al. 2011; Goos et al. 2013; Groh et al. 2012; Madigan et al. 2012) and externalizing problem behavior (Fearon et al. 2010; Hoeve et al. 2012). Fortunately, there is also a vast body of empirical evidence showing that attachment insecurity can be treated effectively (Bakermans-Kranenburg et al. 2003, 2005; Cornell and Hamrin 2008; Wimmer et al. 2009; Zeanah et al. 2005).

To date, however, there is no quick and easy instrument available for providing reliable and valid data on individual child differences in (early) attachment insecurity. Reliable and valid observation instruments that are being used now to assess attachment security are the Strange Situation procedure (Ainsworth et al. 1978) and the Attachment Q-sort (AQS; Van IJzendoorn et al. 2004; Waters and Deane 1985). The Strange Situation (Ainsworth et al. 1978; Main and Solomon 1986, 1990) is considered the ‘golden standard’? to assess attachment in young children. It is a laboratory procedure to assess four patterns of secure (type B) and insecure (type A, C, D) attachment in 1- to 2-year-olds, although an adapted version has been developed to assess attachment in 2 to 5 year olds (Cassidy and Marvin 1992). The caregiver (mostly one of the parents) and child are observed in a room with a one way glass during eight episodes of approximately 3 min each. In episode three, a stranger enters the room, in episode four, the caregiver leaves the child and the stranger alone, in episode five, the caregiver returns and the stranger leaves, in episode six, the caregiver leaves and the child is left to play alone, in episode seven, the stranger returns, and in episode eight, the caregiver returns and the stranger leaves. Throughout the procedure, the child’s responses are observed and coded in order to assess secure, avoidant, ambivalent and disorganized attachment.

An alternative method to validly and reliably assess attachment in young children is the attachment Q-sort (AQS; Van IJzendoorn et al. 2004; Waters and Deane 1985), which is used to observe child attachment outside the laboratory, that is, in the home. The AQS contains 90 cards describing attachment behaviors of children between 1 and 5 years old. A well-trained observer ranks the cards into nine piles from least to most descriptive of the child after several hours of observation. Subsequently, the Q-sort is compared with the Q-sort of a prototypical secure child (rated by experts): the higher the correlation, the more secure the child is, whereas lower scores are indicative of attachment insecurity. In fact, the attachment Q-sort assesses the degree of attachment (in)security instead of the four patterns of attachment that can be derived from the Strange Situation procedure.

Both the Strange Situation procedure and the AQS are time consuming, require intensive training and cannot provide information on sensitivity and specificity (i.e., the proportion of actual positives and negatives that are correctly identified as such, respectively). And, as said, there is no quick and easy instrument available for providing reliable and valid data on individual child differences in (early) attachment insecurity. To fill this gap, the Attachment Insecurity Screening Inventory (AISI) 2–5 years was developed (Polderman and Kellaert-Knoll 2008; Colonnesi et al. 2012). The Attachment Insecurity Screening Inventory is a parent-report questionnaire assessing attachment insecurity of the child. The item pool of the AISI was created by (1) studying recent literature on attachment, (2) examining existing instruments to assess attachment in young children, and (3) conducting interviews with therapists who deliver attachment-based intervention. We argue that both research and clinical practice would benefit from a brief caregiver-report measure to reliably and validly screen for attachment insecurity in pre-school aged children. The AISI 2–5 contains 20 items covering three subscales: avoidant, ambivalent/resistant and disorganized attachment insecurity. The total score of the questionnaire can be considered as an indication of total attachment insecurity.

In the present study, first, the construct validity of the AISI 2–5 was determined by means of a confirmatory factor analysis (CFA). Next, with multi-group CFA, measurement invariance of the instrument was examined across mothers and fathers, as well as across children from a general population sample and a clinical sample (i.e., children referred for clinical treatment of attachment insecurity). Next, reliability information of the total insecurity scale and subscales was examined. Additionally, convergent validity was examined by computing the correlations between the AISI (i.e., caregiver-report of attachment insecurity) and observations of attachment. Also, concurrent validity was studied by computing correlations between attachment, as assessed with the AISI, and both parental sensitivity and psychopathology, and by comparing the AISI scores of children in the general population sample and clinical sample. Moreover, the power of the AISI to discriminate between children with and without attachment insecurity was established by comparing the AISI total difficulty scores of children who were rated secure and insecure on a well-validated measure for observed attachment (AQS), computing the Area Under the Curve (AUC) statistic (Shapiro 1999).

## Method

### Participants

A total of 500 parents (*N* = 500) with 2- to 5-year old children participated in the present study: a general population sample (*n* = 429) and a clinical sample (*n* = 71). The clinical sample consisted of parents with preschoolers, adopted or placed in long-term foster care, who were referred for treatment of attachment insecurity to Basic Trust, which is a group practice of therapists delivering an attachment-based intervention using video interaction guidance (the Basic Trust Method) (Colonnesi et al. 2012; Polderman 1998). The parents in this clinical sample were asked by letter to participate before the treatment started. Parents in the general population sample were recruited through the internet, child care centres, kindergarten, and schools.

### Procedure

In both samples, parents filled in two questionnaires: the Attachment Insecurity Screening Inventory 2–5 years (AISI 2–5 years; Polderman and Kellaert-Knoll 2008) and the Strengths and Difficulties Questionnaire (SDQ; Goodman 2001). Besides, 95 randomly selected parents from the general population sample and 60 parents from the clinical sample were observed at home together with the target child to assess attachment security by means of the attachment Q-sort (AQS; Waters and Deane 1985) and sensitivity by means of the Maternal Behavior Q-sort (MBQS; Pederson et al. 1999) (see below). The observations were carried out by trained research assistants.

The sample of parents who filled in the AISI 2–5 years consisted of 322 mothers and 178 fathers. The mother’s mean age was 35.99 years (*SD* = 4.42) and the father’s mean age was 38.71 years (*SD* = 5.53). The children concerned 268 boys (54 %) and 232 girls (46 %). The mean age of the boys was 3.34 years (*SD* = 1.08) and the mean age of the girls was 3.28 years (*SD* = 1.02). Most parents attended higher professional education (36.6 %), university (31.4 %) or intermediate vocational education (28.4 %). A series of univariate analyses of variance tests and Chi-Square analyses revealed some significant differences between the general population and clinical sample. First, there was an overrepresentation of girls in the clinical sample when compared to the general population sample: *χ*^*2*^(1) = 9.594, *p* = .002. In the general population sample, the child gender distribution was more equal (*n*_boys_ = 242, 56 % boys; *n*_girls_ = 187, 44 % girls) than in the clinical sample (*n*_boys_ = 26, 37 % boys; *n*_girls_ = 45, 63 % girls). Additionally, children in the general sample were somewhat younger (mean age 3.27; *SD* = 1.06) than children in the clinical sample (mean age 3.54; *SD* = 0.98), *F*_(1, 496)_ = 5.172, *p* = .023. Hereby, no significant interaction effects were found between age and gender of the child. Additionally, parents in the general population sample were also somewhat younger (mean age 36.55; *SD* = 4.87) than parents in the clinical sample (mean age 39.44; *SD* = 5.20), *F*_(1, 496)_ = 22.10, *p* = .000. Again, there was no significant interaction effect between age and gender of the parent. In the analyses comparing the means of the general population and clinical sample, we controlled for both gender of the child and age of child and parent. Finally, there was no significant difference in the level of parental education between the general population and clinical sample, *χ*^2^(4) = 2.569, *p* = .632.

### Measures

#### Attachment Insecurity Screening Inventory 2–5 years (AISI)

The AISI parental report 2–5 years contains 20 6-point Likert-type items (never, sometimes, regularly, often, very often, and always) assessing total attachment insecurity by items belonging to three subscales: avoidant, ambivalent/resistant and disorganized attachment insecurity (see the Appendix). The three domains of attachment insecurity symptoms are summed to generate a total score for attachment insecurity.

#### Strengths and Difficulties Questionnaire (SDQ)

The Strengths and Difficulties Questionnaire (SDQ: Goodman 2001; Van Widenfelt et al. 2003) is a brief behavioral screening questionnaire for preschool and school-aged children as well as for adolescents up to and including 16 years of age. The SDQ can be completed by parents, teachers and adolescents and contains 25 items with positive and negative attributes. The respondents used 3-point Likert-type scales to indicate to what extent each attribute applied to the preschool-aged child. With the SDQ four domains of psychopathological symptoms were assessed: emotional symptoms, conduct problems, hyperactivity-inattention and peer problems. The four domains of psychopathological symptoms can also be summed to generate a total score for psychopathology. The validity and reliability of the SDQ have been established by Goodman (2001), who reported satisfactory internal reliability, inter-rater reliability and test-retest reliability in a study among 10.438 British children. Muris et al. (2003) showed that the SDQ is a valid and reliable instrument for diagnosing psychopathology in a sample of 562 Dutch school-aged children. A recent review of the psychometric properties of the parent and teacher report version of the SDQ for children younger than 13 years old, including 48 studies, showed satisfactory reliability and validity of the SDQ (Stone et al. 2010). In the present study, internal consistency reliabilities in terms of Cronbach’s *α* were as follows: emotional symptoms (*α* = .71), conduct problems (*α* = .76), hyperactivity-inattention (*α* = .80), peer problems (*α* = .62), and total difficulties (*α* = .83).

#### Q-sorts for Observed Attachment and Parents’ Sensitivity

Both the Attachment Q-Sort (AQS; Waters and Deane 1985) and the Maternal Behavior Q-sort (MBQS; Pederson et al. 1999) contain 90 items. The Attachment Q-sort (AQS) assesses attachment security of children between 1 and 5 years old (secure-base behavior) and the Maternal Behavior Q-sort (MBQS) assesses parental sensitivity. The procedure for rating attachment corresponds with rating sensitivity. The 90 items are sorted in 9 clusters of items containing 10 items each. Attachment and sensitivity scores are calculated by computing the correlation between the observer sort and a criterion sort of the prototypically secure child (Waters and Deane 1985) or prototypically sensitive parent (Pederson et al. 1999), respectively. The validity of the attachment Q-sort was examined by Van IJzendoorn et al. (2004), who reported satisfactory convergent, discriminant and predictive validity. Evidence of concurrent and predictive validity of the MBQS can be derived from a meta-analysis by Atkinson et al. (2000) and, in an earlier study, Atkinson et al. (1999) reported favorable reliabilities of the MBQS.

In the present study, the AQS and MBQS assessments (*n* = 155, covering the entire age range) were conducted in the home situation by research assistants who had been extensively trained by an expert in attachment. One of the research assistants observed attachment and the other observed sensitivity for both 1 h. The protocol was as follows: greeting and parent–child free play (45 min), brief separation-reunion procedure (5 min), and snack-time (10 min). In the present study, inter-rater reliability of the AQS and MBQS were both sufficient, with ICC = 0.72 and ICC = 0.83, respectively (*n* = 20).

## Results

### Construct Validity

**Fig. 1:**
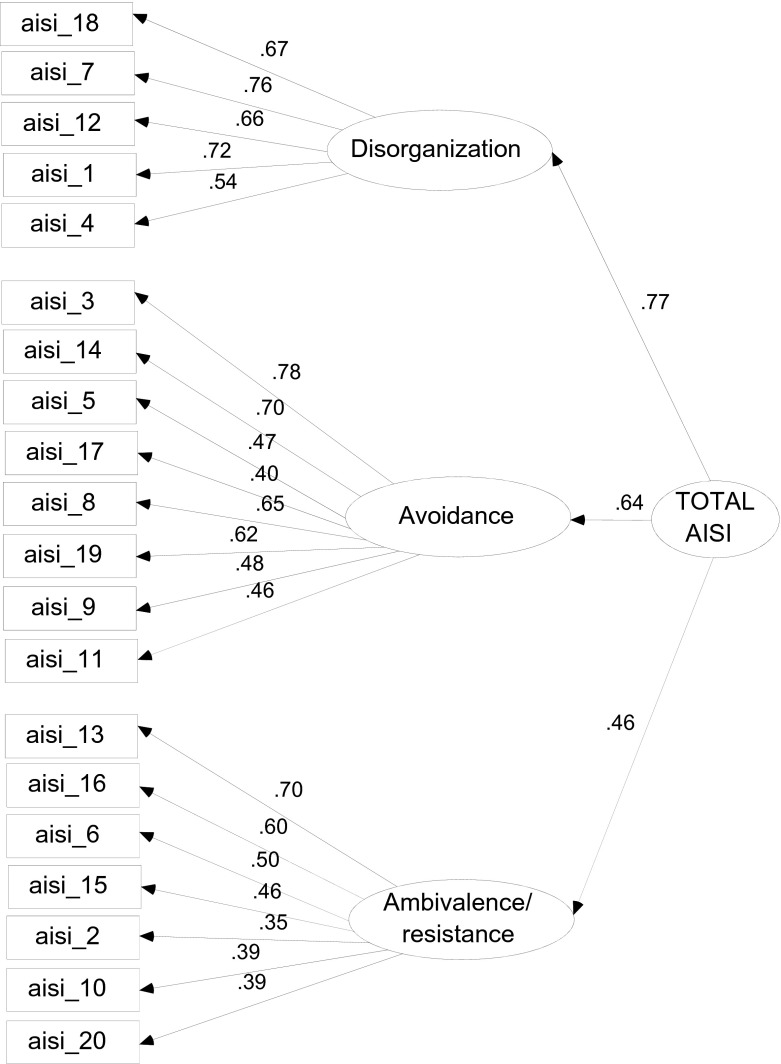
Factor model (CFA) of AISI 2–5 years. *Note.* In the factor model, the error terms between 5 and 17, 3 and 5, and between 14 and both 5 and 17 were allowed to correlate because of similar content

The construct validity of the AISI 2–5 years was examined by means of confirmatory factor analysis (CFA) in Mplus (Muthén and Muthén 1998–2010).^1^ First, the three-factor model (with one higher-order factor) was tested in the complete dataset (*N* = 500). This three-factor model was based on the literature about the three types of insecurely attached children. Modification indices indicated that allowing four correlations between error terms (all of items referring to touching/cuddling and physical contact) resulted in a better fit. The resulting factor model (see Fig. 1) was tested in Mplus: *χ*^2^(163) = 291.299, *p* < .001. Hu and Bentler (1999) describe that several absolute fit indexes can supplement the *χ*^2^-test to indicate how well an a priori model reproduces the sample data (for instance: GFI, AGFI, SRMR, RMSEA). Mplus provides the SRMR (Standardized Root Mean Square Residual) and the RMSEA (Root Mean Square Error of Approximation). For these fit indexes Hu and Bentler (1999) described cut-off values of respectively 0.08 and 0.06. For the CFI, values of above 0.90 indicate reasonable fit, while values above 0.95 indicate good model fit (Hu and Bentler 1999). The results indicated a reasonable to good fit of the tested factor model to the data: SRMR = 0.047 and RMSEA = 0.040, CFI = 0.949.

Next, measurement invariance across the general population and clinical sample of the instrument was tested, with a multi-group CFA (same model as shown in Fig. 1). First, in the constrained model, the factor loadings were constrained to be equal across the two groups (i.e., general population and clinical sample), *χ*^2^(343) = 535.496, *p* < .001. Additional fit indexes again indicated a reasonable to good model fit: SRMR = 0.065 and RMSEA = 0.047, CFI = 0.918. Second, as an alternative model, the unconstrained model (in which the factor loadings were allowed to differ across the two samples) was tested. The comparative fit of the latter model was tested using a *χ*^2^-difference test and the results indicated that the unconstrained (or unequal/measurement variant) model did not lead to a significant improvement in model fit, Δχ^2^(17) = 22.642, *p* = 0.161 and confirm measurement invariance of the instrument across different types of samples (i.e., general population and clinical sample).

In the second place, measurement invariance across the mother and father samples was tested using the same approach. The constrained model was tested first, *χ*^2^(343) = 541.499, *p* < .001, SRMR = 0.063, RMSEA = 0.048, CFI = 0.920. Next, the unconstrained model was tested and compared with the constrained model with the *χ*^2^-difference test, Δχ^2^(17) = 12.047, *p* = .797. In other words, the results of the multi-group CFA also indicated measurement invariance of the instrument across mothers and fathers.

### Reliability

The internal consistency reliabilities in the total sample in terms of Cronbach’s α were satisfactory for the subscale ambivalence/resistance (α = .67) to good for the two other subscales and the total scale: avoidance (α = .80), disorganization (α = .79), total attachment insecurity (α = .81). Additionally, Guttman’s lambda’s 2 were computed as Cronbach’s α is considered to be a systematic underestimation of reliability (Ligtvoet 2011). These Guttman’s lambda 2 statistics were also satisfactory for the subscale ambivalence/resistance (α = .68) to good for all other scales: avoidance (α = .80), disorganization (α = .80), total attachment insecurity (α = .82). To conclude, together, these results indicated the construct validity and reliability of the factor model with three subscales and one higher-order factor of attachment insecurity.

### Convergent Validity: Associations Between Parent-Report of Attachment Insecurity (AISI) and Observations of Attachment Security (AQS)

A bivariate correlation was computed between the parent-report of total attachment insecurity (AISI) and observed attachment security (AQS) in order to examine convergent validity. A significant reverse association between parent-report of attachment insecurity and observed attachment security was considered indicative of convergent validity. As it is possible that the correlation between parent-report of attachment (AISI) and observed attachment (AQS) is inflated by mean differences in attachment between the general and clinical population sample, which were combined to compute the correlations, we computed partial correlations, controlling for the type of sample (general versus clinical population). Significant negative correlations between parent-report of attachment insecurity on the AISI and observed attachment security on the AQS were found for total insecurity (partial *r* = −.29, *p* < .01), disorganization (partial *r* = −.31, *p* < .001), and avoidance (partial *r* = −.21, *p* < .01), but not for ambivalence/resistance (partial *r* = −.10, ns). Since ambivalence/resistance was not significantly (negatively) associated with observed attachment security, evidence for convergent validity should be considered as partial.

### Concurrent Validity: Associations Between Parent-Report of Attachment Insecurity (AISI) and Observations of Parental Sensitivity (MBQS)

The relation between attachment security or attachment insecurity and parental sensitivity is one of the central tenets of attachment theory (Atkinson et al. 2000; De Wolff and Van IJzendoorn 1997; Van IJzendoorn and De Wolff 1997). It is therefore important to examine this relation when establishing concurrent validity of any attachment measure. As in the clinical sample the association between sensitivity and attachment insecurity may (partially) be influenced by past instead of current parental sensitivity, we again computed partial correlations controlling for the type of sample. In the present study, significant negative associations were found between total attachment insecurity (partial *r* = −.28, *p* < .001), avoidant (partial *r* = −.17, *p* < .05), ambivalent/resistant (partial *r* = −.16, *p* < .05), and disorganized (partial *r* = −.28, *p* < .001) and observed parental sensitivity, further supporting the concurrent validity of the AISI.

### Concurrent Validity: Associations Between Parent-Report of Attachment Insecurity (AISI) and of Psychopathology (SDQ)

Additionally, concurrent validity of the AISI 2–5 was examined by computing correlations between the parent-reports of attachment insecurity (AISI) and of psychopathology (SDQ) (see Table 1). As correlations between parent-report of attachment (AISI) and psychopathology (SDQ) can also be inflated by mean differences in attachment and psychopathology between the general and clinical population sample, we computed partial correlations, controlling for the type of sample (general versus clinical population). As expected, significant correlations were found between attachment insecurity and psychopathology, ranging from partial *r* = .09 (between ambivalence and conduct problems and hyperactivity-inattention) to partial *r* = .54 (between disorganization and conduct problems). All correlations were significant. The correlation between total AISI attachment insecurity and total SDQ difficulties was strong (partial *r* = .46, *p* < .001), and larger than the association between secure observed attachment (assessed with the AQS) and total SDQ difficulties (partial *r* = −.25, *p* < .01). The associations between observed attachment security (AQS) and the SDQ subscales were partial *r* = −.04 for emotional problems, partial *r* = −.22 (*p* < .01) for conduct problems, partial *r* = .25 (*p* < .01) for hyperactivity, and partial *r* = −.19 (*p* < .05) for peer problems. The results of these analyses provided additional confirmation of concurrent validity of the AISI.

**Table 1:** Pearson partial correlations between the three subscales and total attachment insecurity (AISI 2–5 years) and psychopathology (SDQ-four subscales and total)

	Emotional symptoms	Conduct problems	Hyperactivity-inattention	Peer problems	Total difficulties
Ambivalence/resistance	.22***	.09*	.09*	.15 ***	.20***
Avoidance	.15**	.28***	.14**	.21***	.29***
Disorganization	.17***	.54***	.0.35***	.27 ***	.49***
Total attachment insecurity	.25***	.43***	.27***	.26***	.46 ***

*N* = 500. * *p* < .05 ** *p* < .01 *** *p* < .001 (one-tailed)

### Concurrent Validity: Differences Between the General and Clinical Population Sample

In order to further examine concurrent validity of the AISI, a multiple analysis of covariance (MANCOVA) was conducted to compare the mean scores on all AISI scales of the clinical and general population sample (see Table 2). In this analysis, because of the differences between the two samples, gender of the child was added as a factor (besides sample), and the ages of both the child and the parents were included as covariates.

**Table 2:** Estimated marginal means and standard errors for differences between the clinical and general population sample on the three subscales and total attachment insecurity (AISI 2–5 years)

	Clinical (*n* = 71)		General population (*n* = 429)	
	*M*	*SE*	*M*	*SE*
Avoidance	16.44 (boys) 18.87 (girls)	0.91 (boys) 0.69 (girls)	15.98 (boys) 14.94 (girls)	0.29 (boys) 0.34 (girls)
Ambivalence/resistance	14.98	0.44	13.39	0.17
Disorganization	16.31	0.47	12.62	0.18
Total attachment insecurity	48.95	1.05	41.46	0.41

The multivariate results of the analysis of variance revealed significant differences between the general and clinical sample, *F*_(3, 492)_ = 18.98, *p* < .01, partial η^2^ = 0.10, observed power (alpha 0.05) = 1.00. The univariate results showed that the expected differences concerned all the subscales and the total attachment insecurity scale: avoidance, *F*_(1, 494)_ = 12.55, *p* < .01, partial η^2^ = .03; ambivalence/resistance, *F*_(1, 494)_ = 11.15, *p* < .01, partial η^2^ = .02; disorganization, *F*_(1, 494)_ = 54.09, *p* < .01, partial η^2^ = .10; and total attachment insecurity, *F*_(1, 494)_ = 43.59, *p* < .01, partial η^2^ = .08.

Additionally, the univariate results indicated a significant interaction effect between sample and gender of child for the subscale avoidance, *F*_(1, 494)_ = 8.23, *p* < .01, partial η^2^ = .02, observed power (alpha 0.05) = 0.82. Therefore, the means for avoidance are shown for boys and girls separately, and these means indicate that parents in the general population sample reported more avoidance for boys, whereas parents in the clinical sample reported more avoidance for girls. Nevertheless, for both boys and girls, parents in the clinical sample reported more avoidance than parents in the general population sample. Overall, all means in Table 2 show that parents of children in the clinical sample consistently reported more attachment insecurity with the AISI (all subscales and total scale) than parents of children in the general population sample. In sum, the results of this analysis indicated concurrent validity of the AISI.

### Discriminating Power

Finally, the AISI needs to discriminate well between children with and without attachment insecurity. In order to be able to examine the discriminating power of the AISI, it was imperative to establish which children actually did and did not have attachment insecurity. For that purpose, the data of the AQS were used. An attachment score lower than 0.33 was considered to reflect attachment insecurity according to a clinical convention suggested by Waters, who developed the attachment Q-sort, and because in normative samples 67 % of the sorts have scores of 0.33 or above (see Howes and Ritchie 1999; Howes et al. 1988; Van Bakel and Riksen-Walraven 2004; Van IJzendoorn et al. 2004). Following this, a total of *n* = 47 children of the 155 observed children were classified as insecure based on the measure of observed attachment (AQS < 0.33), whereas *n* = 108 children were classified as secure (AQS > 0.33).

The discriminating power of the total AISI scale was established by means of Area Under the Curve (AUC) statistics, which are based on graphical plots of true positives (children who were correctly classified as having attachment insecurity) and false positives (children who were incorrectly classified as having attachment insecurity) given the full range of AISI scores on each scale (Receiver Operating Characteristic curves). An AUC statistic of 0.50 indicates that the AISI does not classify children with and without attachment insecurity better than chance alone, whereas an AUC statistic around 0.70 or higher indicates a much better than chance classification (Dolan and Doyle 2000; Shapiro 1999).

Notably, the AUC-value could only be computed for the total attachment insecurity score, assessing total insecurity, because no equivalent observed measures of the three AISI subscales were available. The AUC value for the AISI total attachment insecurity was 0.742, *p* < .001 (95 % CI: 0.655 < AUC < 0.828), which indicates that the total AISI discriminates well between children with and without attachment insecurity. At the optimal total attachment insecurity cut-off score of 46, derived from the ROC-curve, specificity was 74 % and sensitivity 74 %. With a cut-off score of 57, specificity was 90 % and sensitivity 34 %.

## Discussion

With this study the validity and reliability of the Attachment insecurity Screening Inventory (AISI-parental report) 2–5 years was examined in a general and clinical population sample of preschoolers. Regarding the validity of the AISI, Confirmatory Factor Analysis (CFA) yielded a theoretically based factor solution with the three subscales of attachment insecurity (ambivalent/resistant, avoidant, and disorganized attachment insecurity) and one higher-order total attachment insecurity scale that showed a good fit to the data, which indicated support for construct validity. In more descriptive terms, this indicates that the AISI is efficient in covering the complete domain it purports to measure, in this case, attachment insecurity. Additionally, using multi-group confirmatory factor analyses, the instrument measuring this factor model was found to be measurement invariant both across mothers and fathers and across the general and clinical population sample. This means, roughly speaking, that mothers and fathers and parents of children with and without attachment problems seem to interpret or understand the questions of the AISI in a similar way. This is important, because only then it is insightful to compare the different groups (for instance a comparison of mother and father reports of attachment insecurity in attachment research). If there is no support for measurement invariance, it is not clear whether differences in scores are caused by real differences between the groups or by differences between the groups in how they interpret the questions used. In our view, the establishment of clinical norm scores, which are used to discriminate the ‘average’ children from the ‘clinical’ children, is also only a meaningful enterprise when the scores can be compared across the general and clinical sample.

We found partial support for convergent validity in associations between AISI-scores and observed attachment. Concurrent validity was supported by associations between AISI-scores and observed parental sensitivity and parent-reported psychopathology. These results show that the AISI seems to yield an adequate representation of the concept that it is intended to measure. Finally, the AISI discriminated sufficiently well between children in the general and clinical population sample, and between children rated as secure and insecure on a well-validated measure of observed attachment, yielding an AUC-value of 0.742 for the total AISI insecurity scale. In other words, these findings provide preliminary support for the use of the AISI as a screening instrument for the presence of attachment insecurity problems in pre-schoolers.

All subscales and the total AISI scale were also found to be reliable or, put differently, answers to the questions showed consistency. More specifically, the avoidance and disorganization subscales and the total attachment insecurity scale showed good internal consistency reliabilities in terms of both Cronbach’s alpha and Guttman’s lambda 2. The subscale ambivalence/resistance yielded the least reliable scores, but still showed satisfactory reliability. The ambivalence/resistance subscale also showed the least strong associations with parental sensitivity and reports of psychopathology, although still in the expected direction and significant. All other AISI subscales and the total attachment insecurity scale showed stronger associations with parental sensitivity and reports of the child’s psychopathology (all in the expected direction), supporting their strength.

The correlations between the AISI and SDQ scales were generally quite strong, but might have been upwardly biased due to common method bias (i.e., both measures concern parental report questionnaires). Ideally, correlations with observations of psychopathology should have been included to rule out this possibility. Unfortunately, such observations were not available. On the other hand, Conway and Lance (2010) have argued that it is a misconception that because of common method bias relations between variables are necessarily and routinely upwardly biased. Following their recommendations, we would like to add that both the SDQ and AISI seem appropriate, obvious and reliable parental report measures to assess possible difficulties in the child’s development (i.e., psychopathology and attachment insecurity), because parents are widely considered to be the best accessible informants to report on the behavior of their own children in early childhood. Additionally, there was no item-content overlap between the SDQ and AISI suggesting contamination of measures. Finally, significant associations were found between the AISI and observational measures of parental sensitivity (MBQS) and child attachment (AQS) as well, adding to the evidence supporting concurrent and convergent validity, respectively. Therefore, when put together, the results of this study support the validity of the AISI 2–5 years.

As already described, the present study found support for construct and concurrent validity of the AISI, and partial support for convergent validity. We did not, however, assess divergent validity (i.e., indicated by a lack of an association with measures that measure other concepts). Divergent validity is a contentious matter in the validation of attachment instruments, while there is discussion about what is a viable criterion for the examination of divergent validity. An example is the discussion around the association between attachment and temperament. Some have argued that attachment and temperament constitute separate constructs that should not be correlated (Sroufe 1985). Others have argued and demonstrated that (difficult) temperament can be a risk factor for attachment insecurity, for example, by affecting attachment through its effect on parenting (e.g., Van den Boom 1994). To conclude, it was not completely clear whether for instance a measurement of temperament could function as a viable criterion for the examination of divergent validity of the AISI.

Notably, one could argue that high correlations between parental reports of attachment insecurity and psychopathology would indicate lack of divergent validity, especially if not confirmed by a similar pattern of associations between observed attachment and parental reports of psychopathology. First, in this study, in most cases (except for emotional problems) the strength of the relation between parental reports of attachment and psychopathology was also reflected in the strength of the relation between observed attachment and parental reports of psychopathology. Second, the magnitude of the correlations between parent reports of attachment insecurity and psychopathology ranged from small to moderate, with a maximum shared variance of 25 %, which supports divergent validity of the AISI given that most variance in attachment insecurity was not explained by psychopathology. Moreover, a series of recent meta-analyses have shown that effect sizes for the association between attachment and psychopathology are generally medium-to-large, with ambivalence/resistance showing relatively strong associations with emotional symptoms (anxiety/depression) and disorganization showing relatively strong associations with externalizing problems, such as conduct problems (Brumariu and Kerns 2010; Colonnesi et al. 2011; Goos et al. 2013; Groh et al. 2012; Madigan et al. 2012). This pattern of results largely concurs with the distinct associations between the AISI and SDQ subscales, which could be interpreted as support for divergent validity of the AISI.

Some limitations of the current study should be mentioned. First, the AUC-value and sensitivity and specificity statistics of the AISI were based on a relatively small subsample of child-parent dyads who were observed at home (*n* = 155). Ideally, this study should be replicated in a larger and more heterogeneous sample.

With such a future study, one could also further examine possible gender differences in attachment, both as a result of the gender of the child and of the gender of the parent. It should be acknowledged that children’s attachment to mother and father is not necessarily the same (even though they do seem to interpret the AISI questions similarly), as there may be differences between fathers and mothers in parental sensitivity and attachment behaviors. Although fathers and mothers have been found to be equally capable of sensitive responding, fathers tend to exercise their sensitivity less often (Lamb 1982). Moreover, there is growing evidence showing that fathers and mothers react differently to attachment signals in their children. Whereas fathers are prone to support their children in a challenging way, emphasizing autonomy, independency and exploration in their children, mothers tend to be more care-oriented and comforting, emphasizing the attachment pole of the attachment-exploration balance (Bacro and Florin 2009; Grossmann et al. 2002; Noom 1999; Paquette 2004). On the other hand, research findings showed no differences in general attachment insecurity regarding fathers and mothers, which replicates results from meta-analyses of the relation between parental sensitivity and attachment, that did not reveal differences in percentages of (overall) insecure attachment between infant-father and infant-mother dyads, although the relation between sensitivity and attachment proved to be somewhat stronger in mothers than in fathers (De Wolff and Van IJzendoorn 1997; Van IJzendoorn and De Wolff 1997). Additionally, Bakermans-Kranenburg and Van IJzendoorn (2009) conducted a review of child gender differences in child attachment, and only found child gender differences in studies using doll-play narratives instead of in depth interviewing or behavioral observation, with less secure and more avoidant attachments in boys than in girls. They considered these differences to be an artefact caused by differences in verbal abilities between boys and girls, and a contamination of avoidant attachment with ‘macho’ narratives. However, according to Del Giudice (2009), sex differences in attachment do not constitute an artefact of the assessment procedure, since boys and girls could have a different social and genetic propensity to show avoidant (boys) or ambivalent/resistant (girls) attachment. He argues that from a reproductive perspective, at least in times of increased environmental risk, insecure males could best adopt an avoidant strategy, which is associated with little commitment and low investment in parenting, whereas females may take advantage from an ambivalent/ resistant care-eliciting strategy in order to share reproductive costs with their relatives. A further examination of possible differences in attachment as a result of gender of the child and/or parent is a logical next step, following the current study, which was primarily aimed at testing the validity and reliability of the AISI 2–5 years.

A third point is that only the convergent validity of the total AISI insecurity scale could be adequately examined, because no measures of observed attachment insecurity were available that matched the distinctive categories of attachment insecurity measured with the AISI. Conclusions about the convergent validity of the AISI should therefore be considered as preliminary, and interpreted with caution. A future study could use an adapted version of the Strange Situation procedure for use with toddlers in order to be able to establish convergent validity of the AISI subscales of avoidant, ambivalent and disorganized attachment insecurity. Also, observations of psychopathology of the child should ideally be added in future studies to be able to rule out the possibility of common method bias.

Notwithstanding these limitations, the present study does indicate that the AISI is a promising instrument for measuring attachment insecurity in preschoolers in a relatively easy and fast way (20 items; parent-report). The AISI is the first instrument for measuring parental perceptions of child-parent attachment insecurity in children aged 2 to 5 years old that shows satisfactory reliability and sufficient construct and concurrent validity, as well as partial support for convergent validity. Moreover, the results show that the total AISI scale has favorable discriminating power. Therefore, the instrument could play a significant role in scientific research and clinical practice if results are replicated in further studies that independently assess children’s psychopathology, and additional evidence is found for convergent and predictive validity. For scientific research, it could be easier to measure attachment problems in this young age group (2–5 years). This might facilitate, for example, the inclusion of the subject of ‘attachment difficulties’ in the collection of large longitudinal datasets that start at an early age, making longitudinal research and the examination and tracking of attachment problems and associated developmental outcomes possible. Such projects could inform prevention and intervention workers, who wish to provide help as soon as needed. If further studies replicate the current study’s findings, the AISI could be used in practice as a quick screening instrument for attachment difficulties. Further examination of children who score high on the AISI could provide more detailed and contextualized information about the specific problems that children and parents struggle with and offers more exact directions for the assistance that is needed in order to stimulate these children’s positive development.

## Appendix

Attachment Insecurity Screening Inventory (AISI) 2–5 years: 20-item Parental Report (1 = never; 2 = sometimes; 3 = regularly; 4 = often; 5 = very often; 6 = always)

1. Does your child try to force you to do what he/she wants?
2. Is your child excessively docile and obedient?
3. Does your child respond well and remain relaxed when you touch him/her (R)?
4. Does your child stay in control when playing with you?
5. Does your child enjoy being cuddled by you (R)?
6. Does your child cling to you?
7. Does your child argue with you if things do not turn out the way he/she expects?
8. Does your child let you comfort him/her if he/she is in pain, frightened or upset (R)?
9. Does your child ask for help with problems (R)?
10. Is your child over-concerned when you are upset or unwell?
11. Is it easy for your child to resume contact with you after you have been separated (R)?
12. Is your child excessively determined to decide everything for him/herself?
13. Is your child excessively emotional when you leave him/her for a short period of time?
14. Is your child able to enjoy contact with you (R)?
15. Does your child want to be put down and then immediately picked up again?
16. Does your child keep a close eye on you while you do things in and around the house?
17. Does your child hug or cuddle you spontaneously (R)?
18. Does your child become angry with you quickly?
19. Is your child happy and playful in your presence (R)?
20. Does your child need you to reassure him/her that he/she is doing something right?

*Note*. Avoidance subscale items: 3, 5, 8, 9, 11, 14, 17, 19, Ambivalence/resistance subscale items: 2, 6, 10, 13, 15, 16, 20, Disorganization subscale items: 1, 4, 7, 12, 18; (R) = reversely coded.

## Footnotes

1 For this procedure, besides the default estimator, we also made use of the MLR (maximum likelihood with robust standard errors and a chi-square) estimator. This M*plus*-based approach does not require an assumption of normality, because it implements nonnormality robust SE calculations. In other words, the MLR estimator allows relaxation of the normality assumption (Millsap and Maydeu-Olivares 2009, p. 594). The results of the analysis with this MLR estimator also indicated good fit for the tested factor model to the data (even better than the reported results: *χ*^2^(163) = 244.013, *p* = .000; RMSEA = 0.032; SRMR = 0.045). However, as the chi-square value for the MLR cannot be used for chi-square difference tests (Muthén and Muthén 1998-2010), which were needed for the subsequent measurement invariance analyses, we reported the default results in the manuscript. Note thus that these default results seem more robust results or more careful (with a slight underestimation of model fit to the data, i.e., not inflated results).

## References

[CR1] Ainsworth MDS, Blehar MC, Waters E, Wall S (1978). Patterns of attachment: A psychological study of the strange situation.

[CR2] Atkinson L, Chisholm VC, Scott B, Goldberg S, Vaughn BE, Blackwell J, Dickens S, Frances T (1999). Maternal sensitivity, child functioning level and attachment in Down’s Syndrome. Monographs of the Society for Research in Child Development.

[CR3] Atkinson L, Niccols A, Paglia A, Coolbear J, Parker KCH, Poulton L, Guger S, Sitarenios G (2000). A meta-analysis of time between maternal sensitivity and attachment assessments: Implications for internal working models in infancy/toddlerhood. Journal of Social and Personal Relationships.

[CR4] Bacro F, Florin A (2009). La relation père-enfant, la nature et l’organisation des relations d’attachment [The father-child relationship, the nature and organisation of attachment relationships]. Canadian Psychology.

[CR5] Bakermans-Kranenburg MJ, Van IJzendoorn MH (2009). No reliable gender differences in attachment across the life span. Behavioral and Brain Sciences.

[CR6] Bakermans-Kranenburg MJ, Van IJzendoorn MH, Juffer F (2003). Less is more: meta-analysis of sensitivity and attachment interventions in early childhood. Psychological Bulletin.

[CR7] Bakermans-Kranenburg MJ, Van IJzendoorn MH, Juffer F (2005). Disorganized infant attachment and preventive interventions: a review and meta-analysis. Infant Mental Health Journal.

[CR8] Bowlby J (1988). A secure base: Clinical applications of attachment theory.

[CR9] Brumariu LE, Kerns KA (2010). Parent–child attachment and internalizing symptoms in childhood and adolescence: a review of empirical findings and future directions. Development & Psychopathology.

[CR10] Cassidy, J., & Marvin, R. S. (1992). *Attachment in preschool children: Coding guidelines*. Unpublished coding manual, MacArthur Working Group on Attachment, Seattle, WA.

[CR11] Colonnesi C, Draijer EM, Stams GJJM, Van der Bruggen CO, Bögels SM, Noom MJ (2011). The relation between insecure attachment and child anxiety: a meta-analytic review. Journal of Clinical Child & Adolescent Psychology.

[CR12] Colonnesi C, Wissink IB, Noom MJ, Asscher JJ, Hoeve M, Stams GJJM, Polderman N, Kellaert-Knol MG (2012). Basic trust: an attachment-oriented intervention based on mind-mindedness in adoptive families. Research on Social Work Practice.

[CR13] Conway JM, Lance CE (2010). What reviewers should expect from authors regarding common method bias in organizational research. Journal of Business and Psychology.

[CR14] Cornell T, Hamrin V (2008). Clinical interventions for children with attachment problems. Journal of Child and Adolescent Psychiatric Nursing.

[CR15] De Wolff MS, Van IJzendoorn MH (1997). Sensitivity and attachment: a meta-analysis on parental antecedents of infant attachment. Child Development.

[CR16] Del Giudice M (2009). Sex, attachment, and the development of reproductive strategies. Behavioral and Brain Sciences.

[CR17] Dolan M, Doyle M (2000). Violence risk prediction. Clinical and actuarial measures and the role of the Psychopathy Checklist. British Journal of Psychiatry.

[CR18] Dozier M, Stovall K, Albus KE, Bates B (2001). Attachment for infants in foster care: the role of caregiver state of mind. Child Development.

[CR19] Fearon RP, Bakermans-Kranenburg MJ, Van IJzendoorn MH, Lapsley A, Roisman GI (2010). The significance of insecure attachment and disorganization in the development of children’s externalizing behavior: a meta-analytic study. Child Development.

[CR20] Goodman R (2001). Psychometric properties of the Strengths and Difficulties Questionnaire. Journal of the American Academy of Child and Adolescent Psychiatry.

[CR21] Goos, M. J. A., Weenink, N. J., Stams, G. J. J. M., Colonnesi, C., Meijer, A. M., & Rodenburg, H. R. (2013). *The relation between insecure attachment and depression in children and adolescents: A meta-analysis.* Manuscript submitted for publication. 10.1007/s10567-019-00299-9 PMC7000490 31392452

[CR22] Groh AM, Roisman GI, Van IJzendoorn MH, Bakermans-Kranenburg MJ, Fearon P (2012). The significance of insecure and disorganized attachment for children’s internalizing symptoms: a meta-analytic study. Child Development.

[CR23] Grossmann K, Grossmann E, Fremmer-Bombik E, Kindler H, Scheuerer-Englisch H, Zimmermann P (2002). The uniqueness of the child-father attachment relationship: fathers’ sensitive and challenging play as a pivotal variable in a 16-year longitudinal study. Social Development.

[CR24] Hoeve M, Stams GJ, Van der Put CE, Dubas JS, Van der Laan PH, Gerris JR (2012). A meta-analysis of attachment to parents and delinquency. Journal of Abnormal Child Psychology.

[CR25] Howes C, Ritchie S (1999). Attachment organizations in children with difficult life circumstances. Development and Psychopathology.

[CR26] Howes C, Rodning C, Galluzzo DC, Myers L (1988). Attachment and child care: relationships with mother and caregiver. Early Childhood Research Quarterly.

[CR27] Hu L, Bentler PM (1999). Cutoff criteria for fit indexes in covariance structure analysis: conventional criteria versus new alternatives. Structural Equation Modeling.

[CR28] Lamb ME (1982). Paternal influences on early socio-emotional development. Journal of Child Psychology and Psychiatry.

[CR29] Ligtvoet, R. (2011). *Lower bounds to the test reliability*. Retrieved May 2, 2012, from University of Amsterdam, Department of Child Development and Education Website: http://home.medewerker.uva.nl/r.ligtvoet/bestanden/lowerbounds.pdf.

[CR30] Madigan S, Atkinson L, Laurin K, Benoit D (2012). Attachment and internalizing behavior in early childhood: a meta-analysis. Developmental Psychology.

[CR31] Main M, Solomon J, Brazelton TB, Yogman MW (1986). Discovery of an insecure-disorganized/disoriented attachment pattern. Affective development in infancy.

[CR32] Main M, Solomon J, Greenberg MT, Cicchetti D, Cummings EM (1990). Procedures for identifying infants as disorganized/disoriented during the Ainsworth Strange Situation. Attachment in the preschool years: Theory, research, and intervention.

[CR33] Millsap RE, Maydeu-Olivares A (2009). The SAGE handbook of quantitative methods in psychology.

[CR34] Moss E, Rousseau D, Parent S, St-Laurent D, Saintonge J (1998). Correlates of attachment at school age: maternal reported stress, mother–child interaction, and behavior problems. Child Development.

[CR35] Muris P, Meesters C, Van den Berg F (2003). The Strengths and Difficulties Questionnaire (SDQ). European Child & Adolescent Psychiatry.

[CR36] Muthén, L. K., & Muthén, B. O. (1998–2010). *Mplus User’s Guide. Sixth Edition*. Los Angeles, CA: Muthén & Muthén.

[CR37] Noom MJ (1999). Adolescent autonomy: Characteristics and correlates.

[CR38] O’Connor TG, Zeanah CH (2003). Attachment disorders: assessment strategies and treatment approaches. Attachment & Human Development.

[CR39] Paquette D (2004). Theorizing the father–child relationship: mechanisms and developmental outcomes. Human Development.

[CR40] Pederson, D. R., Moran, G., & Bento, S. (1999). *Maternal behaviour Q-sort manual 3.1.* London: University of Western Ontario, Department of Psychology.

[CR41] Polderman N (1998). Hechtingsstoornis, beginnen bij het begin [Attachment disorder: Begin at the beginning]. Tijdschrift voor Orthopedagogiek.

[CR42] Polderman N, Kellaert-Knoll MG (2008). Manual of the attachment insecurity screening inventory 2–5 years (AISI 2–5 years).

[CR43] Shapiro JH (1999). The interpretation of diagnostics tests. Statistical Methods in Medical Research.

[CR44] Sroufe LA (1985). Attachment classification from the perspective of infant-caregiver relationships and infant temperament. Child Development.

[CR45] Stone LL, Otten R, Engels RCME, Vermulst AA, Janssens JMAM (2010). Psychometric properties of the parent and teacher versions of the Strengths and Difficulties Questionnaire for 4-to 12-year-olds: a review. Clinical Child and Family Psychology Review.

[CR46] Vallance DD (2004). Using theory and research on controlling attachments to inform the clinical assessment of pre-school children. Clinical Child Psychology and Psychiatry.

[CR47] Van Bakel HJA, Riksen-Walraven JM (2004). AQS security scores: what do they represent? A study in construct validation. Infant Mental Health Journal.

[CR48] Van den Boom D (1994). The influence of temperament and mothering on attachment and exploration. Child Development.

[CR49] Van IJzendoorn MH, De Wolff MS (1997). In search of the absent father meta-analyses of infant-father attachment: a rejoinder to our discussants. Child Development.

[CR50] Van IJzendoorn MH, Schuengel C, Bakermans-Kranenburg MJ (1999). Disorganized attachment in early childhood: meta-analysis of precursors, concomitants, and sequelae. Development and Psychopathology.

[CR51] Van IJzendoorn MH, Vereijken CMJL, Bakermans-Kranenburg MJ, Riksen-Walraven JM (2004). Assessing attachment security with the attachment Q-sort: meta-analytic evidence for the validity of the observer AQS. Child Development.

[CR52] Van Widenfelt BM, Goedhart AW, Treffers PDA, Goodman R (2003). Dutch version of the Strengths and Difficulties Questionnaire (SDQ). European Child & Adolescent Psychiatry.

[CR53] Waters E, Deane K (1985). Defining and assessing individual differences in attachment relationships: Q-methodology and the organization of behavior in infancy and early childhood. Monographs of the Society for Research in Child Development.

[CR54] Wimmer JS, Vonk ME, Bordnick P (2009). A preliminary investigation of the effectiveness of attachment therapy for adopted children with reactive attachment disorder. Child and Adolescent Social Work Journal.

[CR55] Zeanah, C. H., Smyke, A. T., & Koga, S. (2005). *The Bucharest Early Intervention Project: Attachment and disorders of attachment.* Paper presented at the biennial meeting of the Society for Research in Child Development, Atlanta, GA.

